# Posterior minimal extrathyroidal extension as an independent risk factor for lateral lymph node metastasis in papillary thyroid carcinoma: a retrospective study based on a nomogram prediction model

**DOI:** 10.3389/fendo.2026.1853174

**Published:** 2026-06-17

**Authors:** Qi Zhao, Tong Li, Haochang Gu, Bin Lv, Nan Liu

**Affiliations:** 1 Department of Thyroid Surgery, General Surgery, Qilu Hospital of Shandong University, Jinan, Shandong, China; 2 Thyroid and Breast Surgery, The Fourth People’s Hospital of Jinan, Jinan, Shandong, China

**Keywords:** extrathyroidal extension, lateral lymph node metastasis, papillary thyroid carcinoma, predictive model, risk factors

## Abstract

**Background:**

Extrathyroidal extension (ETE) is a risk factor for lateral lymph node metastasis (LLNM) in patients with papillary thyroid carcinoma (PTC). However, the different effects of posterior minimal extrathyroidal extension (post-mETE) and anterior minimal extrathyroidal extension (ant-mETE) on LLNM remain unclear. This research aimed to investigate the influence of post-mETE and ant-mETE on LLNM and develop a nomogram to guide perioperative management in PTC patients at high risk for LLNM.

**Methods:**

This retrospective study included consecutive PTC patients treated from December 2017 to March 2025. The identification of clinical and pathological predictive factors for LLNM was conducted using both univariate and multivariate analytical methods, and statistically significant variables were included to construct a predictive nomogram, followed by internal validation.

**Results:**

Among 1798 patients with PTC, 391 cases (21.75%) were pathologically confirmed to have LLNM. Compared to ant-mETE, post-mETE was significantly associated with a higher incidence of LLNM (p<0.001). The results of the multivariate analysis showed that there were 6 independent predictive factors for LLNM: maximum tumor diameter >1 cm (OR = 3.022), bilateral thyroid involvement (OR = 2.198), multifocality of the tumor (OR = 1.374), number of central lymph node metastases ≥2 (OR = 8.083), tumor located at the upper pole (OR = 2.679), and post-mETE (OR = 2.036). The nomogram constructed in this study demonstrated good discrimination performance (AUC = 0.850) and clinical applicability.

**Conclusion:**

Compared to ant-mETE, PTC patients with post-mETE were more likely to develop LLNM. The predictive nomogram demonstrated high accuracy in forecasting LLNM and facilitated the identification of occult lymph node metastases. This capability assisted in determining the need for adjuvant radioactive iodine therapy or stricter follow-up management.

## 1 Introduction

Papillary thyroid carcinoma (PTC) represents the predominant form of thyroid malignancy, comprising more than 80% of all thyroid cancer diagnoses (1). Although its biological behavior is generally characterized as indolent, the incidence of lymph node metastasis is relatively high, with lateral lymph node metastasis (LLNM) in the lateral neck region (levels II-V) significantly associated with the risk of recurrence and survival in patients (2–4). Studies have shown that the presence of LLNM increases the 5-year recurrence risk for PTC patients by 2–3 times and is closely related to the rise in distant metastasis and disease-specific mortality (5, 6). Occult lateral lymph node metastasis (OLLNM) refers to lymph node metastasis in the lateral neck region that is not detected by routine preoperative imaging but is confirmed by postoperative pathology or more sensitive examinations, which is an important cause of postoperative recurrence and secondary surgery. In PTC patients with no obvious LLNM found during preoperative imaging assessment (cN0), the overall incidence of OLLNM varies across different studies, typically reported between 10% and 51.7% (7–9). Among clinically positive LLNM (cN1b) PTC patients but clinically negative level II PTC patients, up to about 35% still have OLLNM in level II (5, 10).

Currently, the risk factors for LLNM in PTC are not fully elucidated. Multiple studies suggest that clinical pathological parameters such as male (OR = 1.51), tumor diameter >2 cm (OR = 3.88), extrathyroidal extension (ETE) (OR = 4.16), central lymph node metastasis (CLNM) (OR = 5.38), and tumors located at the upper pole (OR = 2.14) are independent predictive factors (11, 12). ETE refers to the direct infiltration of surrounding tissues by primary tumor, which can be classified based on the degree of invasion into minimal extrathyroid invasion (mETE) and gross extrathyroid invasion (gETE). For gETE, the criteria involve macroscopic tumor invasion into adjacent structures, including the trachea, esophagus, larynx, recurrent laryngeal nerve, subcutaneous tissue, strap muscles, and, in advanced cases, encasement of major cervical vessels or invasion of the prevertebral fascia (13). In contrast, the criteria for mETE are defined by microscopic evidence of tumor breaching the thyroid capsule and invasion into the sternothyroid muscle or the surrounding soft tissues (14, 15). mETE can be predicted via preoperative ultrasound examination (16), but its definitive diagnosis relies on postoperative histopathological examination.

Based on the direction of invasion, mETE can be further divided into posterior minimal extrathyroid invasion (post-mETE) and anterior minimal extrathyroid invasion (ant-mETE). Whereas, there are currently few studies on whether there are differences in the impact of post-mETE and ant-mETE on LLNM in PTC patients. Therefore, this research sought to explore the distinct impacts of post-mETE and ant-mETE on LLNM and to further investigate the risk factors associated with LLNM in patients with PTC. At the same time, it would construct predictive models to provide references for postoperative risk assessment of LLNM and individualized treatment decisions in clinical practice.

## 2 Patients and methods

### 2.1 Study subjects

**Figure 1:**
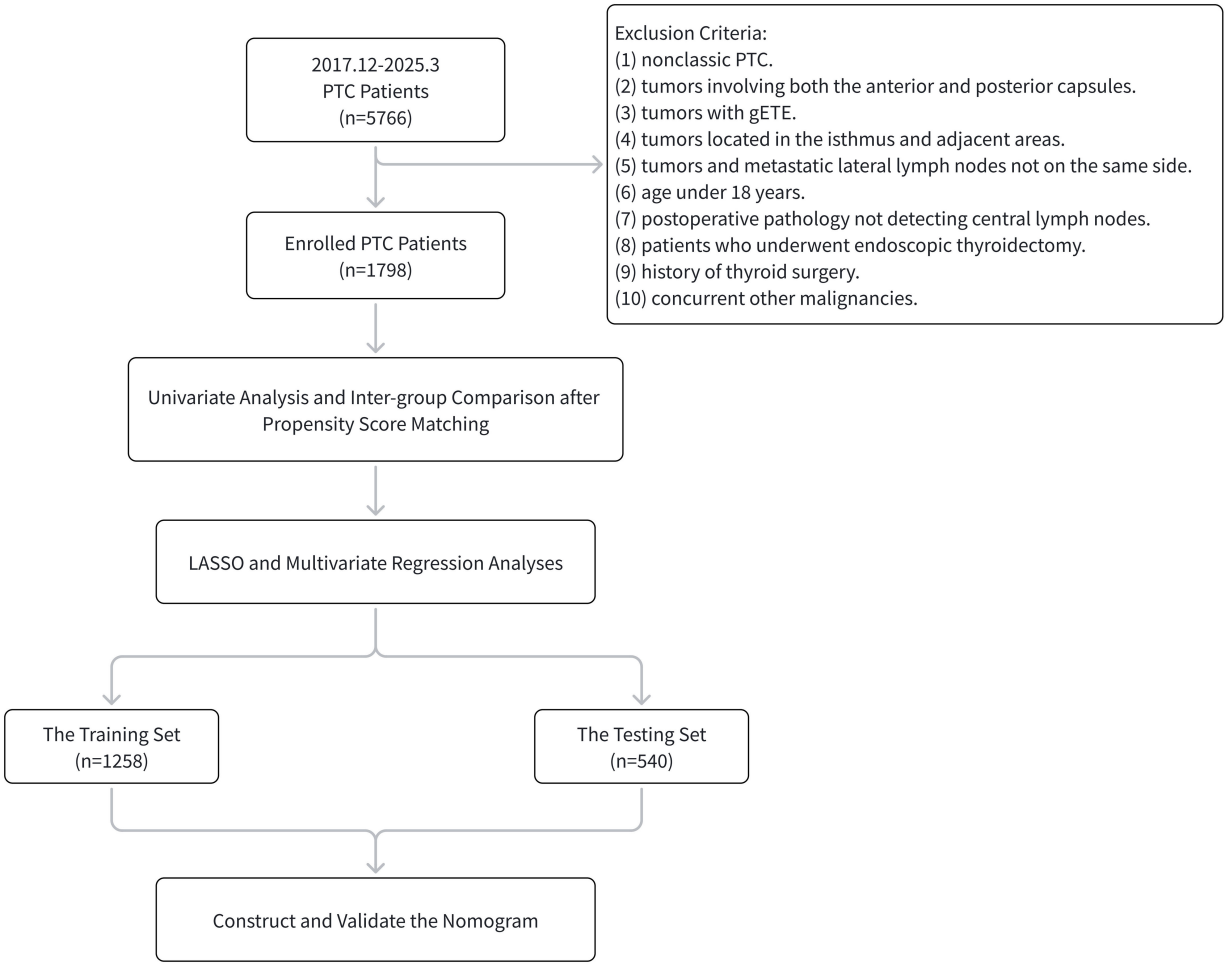
The flowchart of study design.

This study conducted a retrospective analysis of 5766 patients with PTC who underwent their first thyroid surgery at Qilu Hospital of Shandong University from December 2017 to March 2025. Cases were excluded based on the following criteria: (1) nonclassic PTC; (2) tumors involving both the anterior and posterior capsules; (3) tumors with gETE; (4) tumors located in the isthmus and adjacent areas; (5) tumors and metastatic lateral lymph nodes not on the same side; (6) age under 18 years; (7) postoperative pathology not detecting central lymph nodes; (8) patients who underwent endoscopic thyroidectomy; (9) history of thyroid surgery; (10) concurrent other malignancies. Patients were analyzed and categorized into positive and negative groups based on LLNM. The method for grouping patients was as follows: (1) patients without suspicious lateral lymph nodes indicated by preoperative ultrasound were not dissected lateral lymph nodes intraoperatively and classified as the negative group; (2) patients with suspicious metastatic ultrasound signs (strong echoes, cystic changes) confirmed by fine needle aspiration or thyroglobulin (Tg) testing underwent lateral lymph nodes dissection (levels II-V) and were categorized as positive or negative according to postoperative pathology; (3) patients with suspicious metastasis indicated by preoperative ultrasound but refused diagnostic examination underwent regional dissection based on ultrasound and frozen pathology, and if metastasis was confirmed, lateral lymph node dissection was performed, ultimately classified based on paraffin pathology. All patients underwent unilateral lobectomy or total thyroidectomy combined with central lymph node dissection (including prelaryngeal, pretracheal, and paratracheal lymph nodes). This research received approval from the Ethics Committee of Qilu Hospital of Shandong University (Approval No. 2018149). The study details were comprehensively communicated to participants both in writing and orally, and authorization for medical record review was obtained. The specific research process was shown in **Figure 1**.

### 2.2 Data collection

This study collected the following clinical pathological parameters of the patients: age, sex, body mass index (BMI), tumor size, lesion distribution features (bilateral or unilateral), multifocality, presence of nodular goiter (NG) or Hashimoto’s thyroiditis (HT), tumor site, mETE, BRAF V600E mutation status, and CLNM status. For age grouping, the American Joint Committee on Cancer (AJCC) 8th edition cancer staging guidelines were referenced, using 55 years as the cutoff point (13). BMI grouping was based on the World Health Organization (WHO) standards, with a critical value of 25 kg/m² (17). Tumor size was recorded based on the greatest diameter of the lesion. Postoperative pathological examination confirmed bilateral lesions, multifocality, HT, NG, and lymph node metastasis. The location of the tumor was categorized into the lower pole, upper pole and middle region based on preoperative ultrasound localization and intraoperative exploration results. If multiple lesions were present, the location of the largest tumor was used.

Preoperative ultrasound examination was first used for screening of mETE. Patients with clear evidence of capsule disruption or tumor protrusion were classified into the mETE group. Patients with unclear capsule rupture recorded in the ultrasound report were assigned in the mETE group following a double-blind discussion and consensus reached by two experienced ultrasound physicians. All mETE were ultimately confirmed based on histopathological results. At the pathological level, mETE refers to microscopic evidence of tumor breaching the thyroid capsule and invasion into the sternothyroid muscle or the surrounding soft tissues.

### 2.3 Statistical methods

Statistical analyses and graphical visualizations were conducted utilizing R software version 4.4.1 (http://www.r-project.org). The pre-set level of statistical significance for this research was two-sided P<0.05. In univariate analysis, comparisons of categorical variables were completed using the chi-square test or Fisher’s exact probability method. Non-normally distributed continuous variables were presented as median (interquartile range) and analyzed using the Mann-Whitney U test and the Kruskal-Wallis H test. To reduce selection bias in non-randomized controlled studies and control confounding factors, a 1:1 propensity score matching method was used to balance other variables in multiple group comparisons, with a matching tolerance set at 0.02. Candidate variables that were statistically significant in univariate analysis were subjected to dimensionality reduction using LASSO regression. The tuning parameter λ was selected via 20-fold cross-validation. To avoid overfitting and ensure model parsimony, the final model variables were determined using λ.1se, resulting in the selection of six variables with non-zero coefficients. After selection through LASSO analysis, the included variables were further introduced into a multivariate logistic regression framework to determine independent predictors. Subsequently, these predictive indicators were integrated to construct a nomogram model. The study cohort was stratified and randomly grouped according to a ratio of 70% for the training set and 30% for the testing set. The model validation was performed from three aspects: evaluating model discrimination using receiver operating characteristic (ROC) curve; verifying model calibration via comparison plots of predicted versus observed probabilities; and measuring the clinical net benefit of the model using decision curve analysis (DCA).

## 3 Results

### 3.1 Baseline characteristics of PTC patients

A total of 1798 patients with PTC were included in this study, and their clinicopathologic characteristics were recorded in **Table 1**. Among them, 391 patients (21.75%) had LLNM, while 1407 patients (78.25%) did not have metastasis. Among the 901 patients with CLNM, 330 (36.63%) patients had concurrent LLNM. However, among the 897 patients without CLNM, 61 (6.80%) patients still had LLNM. Based on the anatomical location of the tumor, there were 512 cases (28.48%) located at the upper pole and 572 cases (31.81%) located at the lower pole. Additionally, 595 patients (33.09%) had ant-mETE, and 615 patients (34.20%) exhibited post-mETE.

**Table 1:** Clinicopathologic characteristics of PTC patients and univariate analysis of risk factors for LLNM.

		Lateral lymph node metastasis				
Characteristics	Total (n=1798)	Yes (n=391, 21.75%)		No (n=1407, 78.25%)		P value
Age						<0.001
<55	1381	332 (84.91%)		1049 (74.56%)		
≥55	417	59 (15.09%)		358 (25.44%)		
Sex						0.131
Female	1336	279 (71.36%)		1057 (75.12%)		
Male	462	112 (28.64%)		350 (24.88%)		
BMI						0.008
<25	883	169 (43.22%)		714 (50.75%)		
≥25	915	222 (56.78%)		693 (49.25%)		
Maximum Tumor Size						<0.001
≤1cm	1333	188 (48.08%)		1145 (81.38%)		
>1cm	465	203 (51.92%)		262 (18.62%)		
Bilateral						<0.001
Yes	388	145 (37.08%)		243 (17.27%)		
No	1410	246 (62.92%)		1164 (82.73%)		
Multifocality						<0.001
Yes	452	146 (37.34%)		306 (21.75%)		
No	1346	245 (62.66%)		1101 (78.25%)		
HT						0.681
Yes	441	99 (25.32%)		342 (24.31%)		
No	1357	292 (74.68%)		1065 (75.69%)		
NG						0.466
Yes	872	196 (50.13%)		676 (48.05%)		
No	926	195 (49.87%)		731 (51.95%)		
CLNM						<0.001
Yes	901	330 (84.40%)		571 (40.58%)		
No	897	61 (15.60%)		836 (59.42%)		
Number of CLNM	1798	2 (1,5)		0 (0,1)		<0.001
Tumor Location						<0.001
Middle	714	138 (35.29%)		576 (40.94%)		
Upper	512	184 (47.06%)		328 (23.31%)		
Lower	572	69 (17.65%)		503 (35.75%)		
mETE						<0.001
Without	588	64 (16.37%)		524 (37.24%)		
Anterior	595	116 (29.67%)		479 (34.05%)		
Posterior	615	211 (53.96%)		404 (28.71%)		
BRAF V600E Mutation						0.115
Positive	1343	304 (77.75%)		1039 (73.85%)		
Negative	455	87 (22.25%)		368 (26.15%)		

PTC, papillary thyroid carcinoma; LLNM, lateral lymph node metastasis; BMI, body mass index; HT Hashimoto’s thyroiditis; NG, nodular goiter; CLNM, central lymph node metastasis; mETE, minimal extrathyroidal extension. Number of CLNM (non-normally distributed) is presented as median (IQR).

**Table 2** assessed the consistency between ultrasound and pathological diagnosis regarding mETE in this study cohort. The results showed that the sensitivity of ultrasound diagnosis for mETE was 86.94%, specificity was 80.95%, positive predictive value (PPV) was 90.38%, and negative predictive value (NPV) was 75.08%.

**Table 2:** Analysis of consistency between preoperative sonographic mETE and postoperative pathological mETE.

Sonographically detected mETE	Pathologically confirmed mETE	
	Positive	Negative
Positive	1052	112
Negative	158	476

mETE, minimal extrathyroidal extension.

### 3.2 Screening of risk factors

The univariate analysis indicated that patient age, BMI, maximum tumor diameter, bilateral lesions, multifocal features, CLNM, tumor location, and mETE were all identified as risk factors for LLNM (**Table 1**). To further clarify the relationship between mETE and tumor behavior, we compared differences in tumor characteristics among the without mETE, anterior mETE, and posterior mETE groups (**Table 3**). The results showed that posterior mETE was more likely to be associated with CLNM (P < 0.001) and LLNM (P < 0.001), whereas no significant difference was observed among the three groups regarding the number of CLNM (P = 0.462). In addition, differences were also found among the three groups in terms of maximum tumor size and multifocality.

**Table 3:** Correlation analysis between minimal extrathyroidal extension and tumor characteristics.

	Minimal extrathyroidal extension			
Characteristics	Without mETE (n=588, 32.70%)	Anterior mETE (n=595, 33.09%)	Posterior mETE (n=615, 34.20%)	P value
Age				0.288
<55	449 (76.36%)	440 (73.95%)	492 (80%)	
≥55	139 (23.64%)	155 (26.05%)	123 (20%)	
Sex				0.160
Female	444 (75.51%)	458 (76.97%)	434 (70.57%)	
Male	144 (24.49%)	137 (23.03%)	181 (29.43%)	
BMI				0.242
<25	310 (52.72%)	287 (48.24%)	318 (51.71%)	
≥25	278 (47.58%)	308 (51.76%)	297 (48.29%)	
Maximum Tumor Size				<0.001
≤1cm	505 (85.88%)	415 (69.75%)	413 (67.15%)	
>1cm	83 (14.12%)	180 (30.25%)	202 (32.85%)	
Bilateral				0.338
Yes	94 (15.99%)	133 (22.35%)	161 (26.18%)	
No	494 (84.01%)	462 (77.65%)	454 (73.82%)	
Multifocality				0.005
Yes	104 (17.69%)	160 (26.89%)	188 (30.57%)	
No	484 (82.31%)	435 (73.11%)	427 (69.43%)	
HT				0.085
Yes	167 (28.40%)	147 (24.71%)	127 (20.65%)	
No	421 (71.60%)	448 (75.29%)	488 (79.35%)	
NG				0.034
Yes	259 (44.05%)	282 (47.39%)	331 (53.82%)	
No	329 (55.95%)	313 (52.61%)	284 (46.18%)	
Tumor Location				0.213
Middle	257 (43.71%)	232 (38.99%)	225 (36.59%)	
Upper	140 (23.81%)	168 (28.24%)	204 (33.17%)	
Lower	191 (32.48%)	195 (32.77%)	186 (30.24%)	
CLNM				<0.001
Yes	204 (34.69%)	282 (47.39%)	415 (67.48%)	
No	384 (65.31%)	313 (52.61%)	200 (32.52%)	
Number of CLNM	0 (0,1)	0 (0,2)	1 (0,3)	0.462
LLNM				<0.001
Yes	64 (10.88%)	116 (19.50%)	211 (34.31%)	
No	524 (89.12%)	479 (80.50%)	404 (65.69%)	
BRAF V600E Mutation				0.235
Positive	431 (73.30%)	453 (76.13%)	459 (74.63%)	
Negative	164 (26.70%)	150 (23.87%)	141 (25.37%)	

mETE, minimal extrathyroidal extension; BMI, body mass index; HT Hashimoto’s thyroiditis; NG, nodular goiter; CLNM, central lymph node metastasis; LLNM, lateral lymph node metastasis; Number of CLNM (non-normally distributed) is presented as median (IQR).

**Figure 2:**
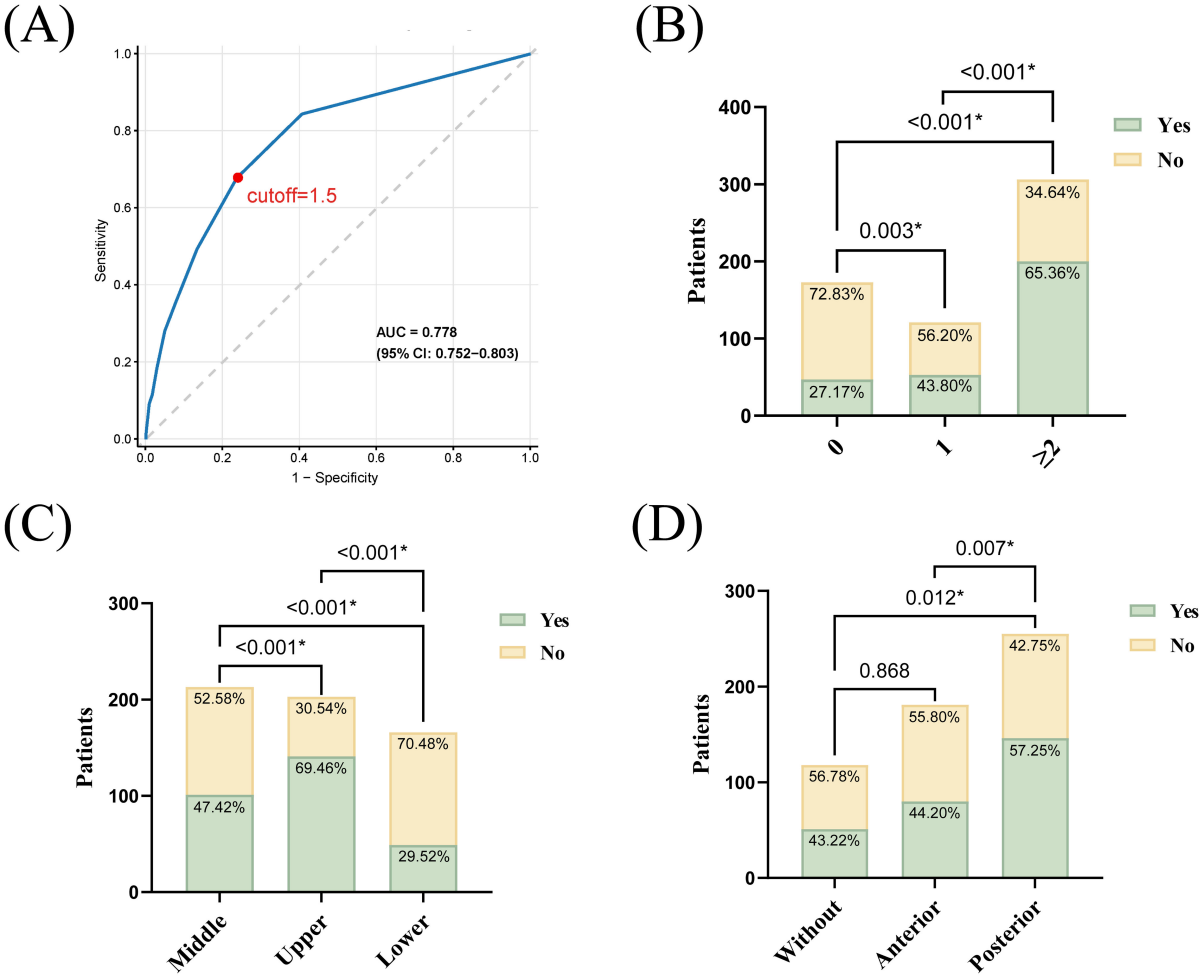
ROC curve for predicting LLNM based on the number of CLNM and intergroup analyses after propensity score matching. **(A)** ROC curve for predicting LLNM based on the number of CLNM and an optimal cutoff value. **(B)** The results among the three subgroups based on CLNM. **(C)** The results among the three subgroups based on tumor location. **(D)** The results among the three subgroups based on mETE.

The ROC curve demonstrated that the number of CLNM had good discriminatory ability for LLNM (AUC = 0.778), with an optimal cutoff value of 1.5 (**Figure 2A**). Therefore, subsequent analyses grouped CLNM by the number of metastases: 0, 1, and ≥2. After propensity score matching, CLNM, tumor location, and mETE remained risk factors for LLNM (**Supplementary Tables 1**–**3**). Analysis of the three subgroups based on the number of CLNM showed that patients with more than 2 metastatic lymph nodes had an increased probability of LLNM (**Figure 2B**). The subgroup analysis by tumor location revealed notable differences in the influence on LLNM across any two groups, with upper pole and middle portion tumors exhibiting a greater propensity to result in LLNM relative to lower pole tumors (**Figure 2C**). In the subgroup comparison based on mETE typing, the risk of LLNM was markedly elevated for post-mETE compared to ant-mETE (**Figure 2D**).

**Figure 3:**
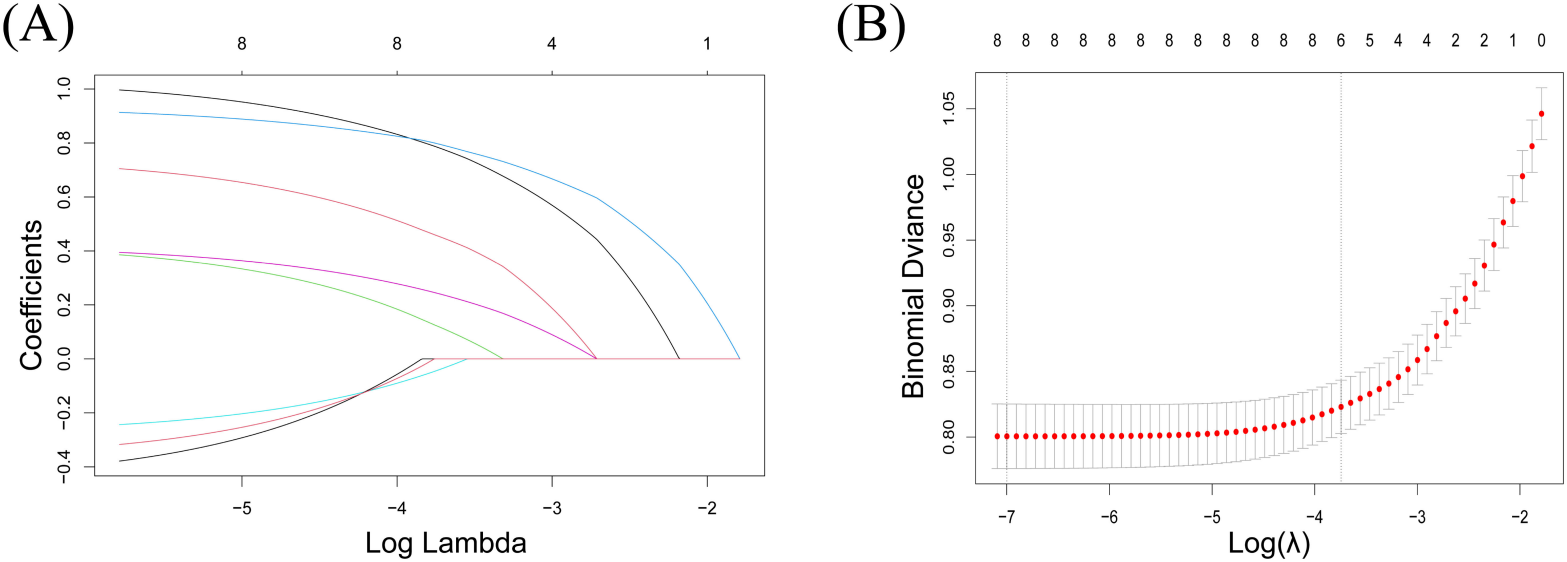
Characteristics selection using the LASSO. **(A)** LASSO coefficient profiles of the risk factors found in univariate logistic regression analysis. **(B)** The results of the cross-validation determined the optimal value of the penalty parameter λ, six independent risk factors for predictive model were identified.

**Figure 4:**
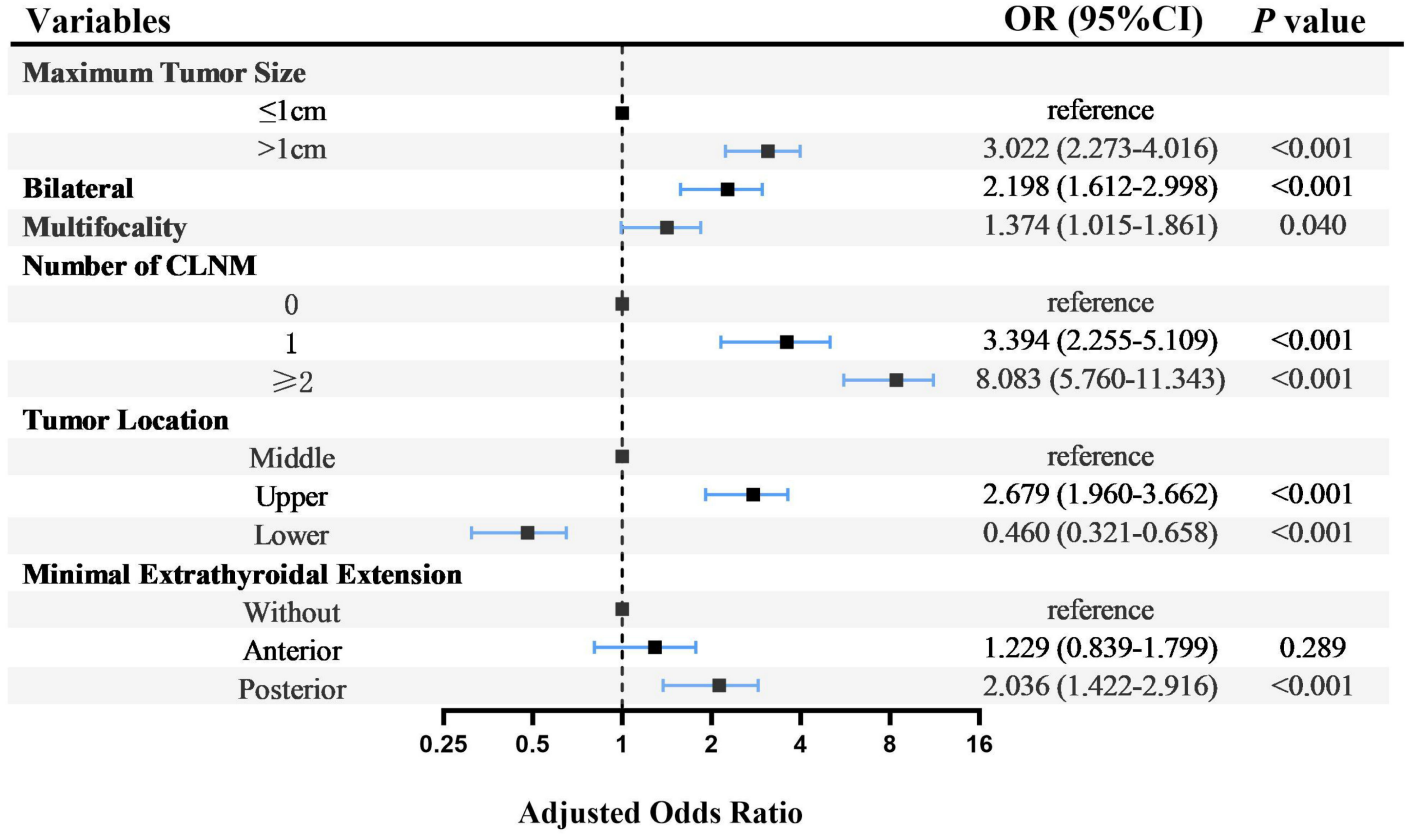
Forest plot of multivariate logistic regression analysis.

To control for potential confounding factors, this study employed a LASSO regression model with regularization constraints. **Figure 3A** showed the coefficient trajectory of the variable selection process, and the optimal penalty parameter λ was determined through 20-fold cross-validation. Using this sparse modeling method, 6 predictors were finally selected: maximum tumor diameter, bilaterality, multifocality, number of CLNM, tumor location, and mETE (**Figure 3B**). Multivariate regression analysis confirmed that these 6 variables were independent risk factors for LLNM (**Figure 4**). Specifically, compared to middle portion tumors, upper pole tumors significantly increased the risk of LLNM (OR = 2.679, 95%CI=1.960-3.662, P<0.001). Furthermore, patients with post-mETE had a higher risk of LLNM than those without mETE (OR = 2.036, 95%CI=1.422-2.916, P<0.001), but ant-mETE was not an independent risk factor for LLNM (OR = 1.229, 95%CI=0.839-1.799, P = 0.289).

### 3.3 Construction and validation of the nomogram

**Figure 5:**
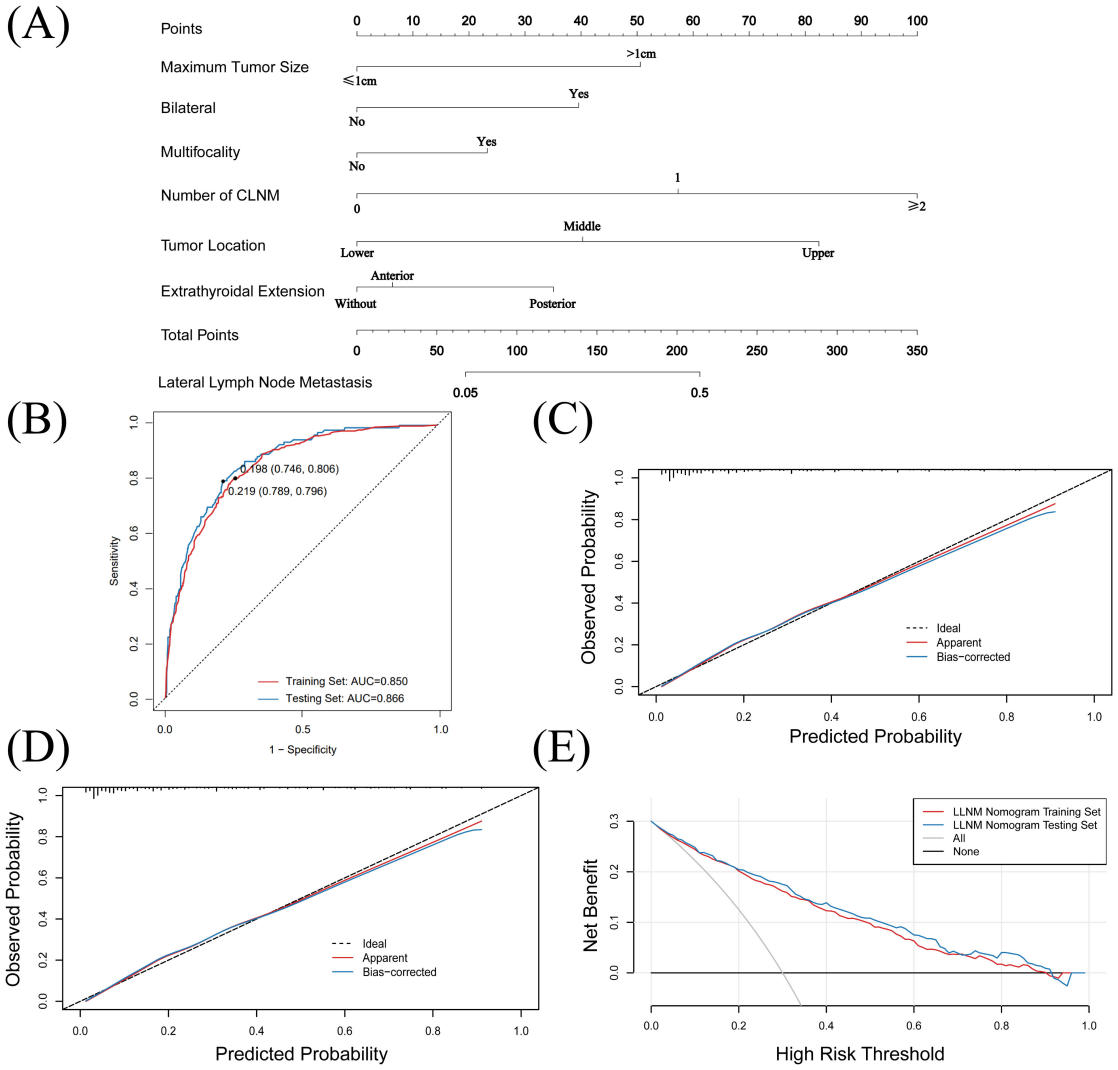
Clinical predictive nomogram for risk of LLNM was constructed and validated. **(A)** Nomogram to predict LLNM in PTC patients. **(B)** ROC curve of nomogram in the training set and the testing set. **(C)** Calibration curves of nomogram for predicting LLNM in the training set. **(D)** Calibration curves of nomogram for predicting LLNM in the testing set. **(E)** Decision curve analysis of nomogram in PTC patients.

To enhance the applicability of the model in diverse clinical populations, this study employed a holdout validation framework. The dataset was divided into a training set (70%, n=1258) and a testing set (30%, n=540) through stratified randomization. A nomogram was developed to assess the likelihood of LLNM according to the six risk factors for LLNM identified through multivariate regression analysis (**Figure 5A**). The nomogram indicated that the number of CLNM contributed most significantly to the risk of LLNM, followed by tumor location. However, mETE did not significantly contribute to the risk of LLNM in the nomogram. The ROC curve indicated that the nomogram model had high predictive efficacy, with AUC of 0.850 (95% CI = 0.826-0.874) for the training set and 0.866 (95% CI = 0.830-0.901) for the testing set (**Figure 5B**). The sensitivity and specificity were 0.81 and 0.75 for the training set, and 0.80 and 0.79 for the testing set. The PPV was approximately 50% for both datasets, while the NPV exceeded 90%, and the accuracy was 80%. For both the training and testing sets, strong agreement between model-predicted probabilities and observed values were found in the calibration curve, reflecting small systematic prediction bias in the model (**Figures 5C, D**). DCA results suggested that clinical interventions guided by this predictive model could yield high net benefits, and using this model to assess patients’ LLNM status would help optimize clinical decision-making (**Figure 5E**).

## 4 Discussion

As the most prevalent type of thyroid cancer, PTC has a continuously rising incidence and has become an important public health issue worldwide. Epidemiological monitoring data in China show that the annual average diagnosis growth rate of PTC reaches 19.8% (18). Although the overall prognosis for PTC patients is good and the long-term survival rate is high, LLNM significantly increases the risk of disease recurrence and treatment complexity and brings higher treatment risks and economic burdens. Therefore, exploration of the risk factors for LLNM is of great clinical significance for accurately predicting disease progression and optimizing personalized treatment plans. This research demonstrated that the maximum tumor diameter, multifocality, bilateral lesions, tumor location, the number of CLNM, and post-mETE were independent risk factors for LLNM. Interestingly, the study found that compared to ant-mETE, post-mETE was more likely to trigger LLNM, and that upper pole tumors carried a higher risk of LLNM compared to those in the middle region and lower poles.

ETE is widely regarded as one of the predictive factors for LLNM. Studies have shown that approximately 30% of differentiated thyroid cancers exhibit ETE (19). Multiple studies have confirmed that ETE is an independent risk factor for CLNM and LLNM in PTC (20–22). ETE raises the risk of tumors breaching local barriers and invading lymphatic and blood vessels, and it is often accompanied by other invasive features. For example, in diffuse sclerosing PTC, ETE has been identified as an independent risk factor for LLNM (23). Some studies suggest that ETE reflects the enhanced ability of tumor cells to breach the basement membrane, indicating active remodeling of the tumor microenvironment and epithelial-mesenchymal transition (EMT) processes (24). This study also confirmed a significant correlation between mETE and LLNM, particularly for posterior mETE. Studies have shown a significant association between high lymphatic vessel density and LNM (25). Therefore, we hypothesize that when anterior mETE occurs, the tumor is covered by the anterior strap muscles, with limited peritumoral lymphatic and adipose tissue, and is located farther from the paratracheal lymph nodes. In contrast, when posterior invasion occurs, the posterior aspect of the thyroid is covered only by a thin layer of loose connective tissue, with a rich lymphatic network, and the posterior capsular lymphatic vessels are directly connected to the paratracheal lymph nodes. This may increase the risk of CLNM and subsequently elevate the risk of LLNM. This suggests that in clinical practice, when assessing ETE, even mETE, the direction of invasion may be more important than whether it occurs.

In recent years, the correlation between the vertical distribution of tumors and lymph node metastasis has also become a research focus. Some studies provide evidence that the risk of CLNM is significantly higher in lower pole tumor (26, 27). Tumor location also affects the pattern of LLNM, with the upper pole tumors being more likely to develop LLNM (OR = 6.78) (28). After adjusting for confounding factors such as tumor size, multifocality, and ETE status, this research also found that compared to middle region tumors, the risk of LLNM in upper pole tumors increased by 2.679 times, while the risk of LLNM in lower pole tumors was significantly reduced. This phenomenon may be related to the anatomical characteristics of lymphatic drainage from the upper pole directly into the internal jugular vein chain.

The number of CLNM is an important indicator for predicting LLNM in PTC. CLNM serves as an important predictor to LLNM, indicating the classic metastatic pattern of tumor cells spreading stepwise along the lymphatic pathways, with mechanisms involving interactions between lymphatic endothelium and tumor cells (29). Multiple researches have shown that when the number of CLNM is more than three, the risk of LLNM significantly increases by 3–5 times (30). This study found that when patients had more than 2 metastatic lymph nodes in the central region, the risk of LLNM was 8 times that of patients without CLNM. This phenomenon may be related to the increased invasiveness of the tumor and the extensive involvement of lymphatic drainage pathways. In addition, metastatic lymph nodes larger than 3 cm and extranodal invasion are also independent predictors of LLNM (31, 32). This may be due to a larger tumor burden and the direct spread of the tumor through lymphatic or blood vessels to the lateral neck region.

This study found that a tumor >1 cm, bilateral lesions, and multifocality were independent risk factors for LLNM. Increased tumor size is usually accompanied by stronger invasive capabilities, which may promote lymphatic invasion by enhancing changes in tumor cell adhesion molecule expression and matrix degrading enzyme activity, further leading to lymph node metastasis (33). Studies show that when the tumor diameter is >2cm, the risk of LLNM significantly increases (OR = 5.408) (34), with each additional centimeter increasing the risk by approximately 30% (35). Bilaterality and multifocality reflect the heterogeneity and multi-origin growth of tumors, which may be related to the diversification of tumor stem cell populations and the accumulation of genetic variations, promoting the activation of multiple pathways for tumor cell metastasis (36). Multifocal lesions increase the risk of lymph node metastasis by 1.5–2 times, with the combined OR value reaching 2.05 (34, 37). Notably, the lymph node metastasis rate for bilateral multifocal lesions (65.27%) is significantly higher than for unilateral lesions (38), and it is independently associated with a higher risk of distant metastasis (HR = 4.77) (39).

In this study, although univariate analysis suggested that age and BMI were also risk factors for LLNM, both did not enter the final predictive model after LASSO selection. Age-related tumor biological characteristics, immune function, and endocrine changes may provide a favorable microenvironment for tumor metastasis (40). Multiple studies have shown that the lymph node metastasis rate in younger patients (<45 years) is significantly higher than in older patients, but the prognosis is relatively better (35, 41). This age difference may be related to the more active metabolism of thyroid tissue and rich lymphatic drainage in younger patients. Obesity may promote tumor invasion and metastasis through certain obesity-related factors and metabolic pathways, such as leptin and adiponectin (42).

This study employed rigorous statistical analysis methods to establish a risk prediction model for LLNM based on nomogram. Nomogram, as a visual predictive tool, has the most ideal predictive efficacy (43) and have been widely applied in the prognostic assessment of various cancers (44, 45). Some studies also have integrated ultrasound features (such as tumor size, margins, calcification) and clinical characteristics (such as age, sex) to construct nomograms for preoperative prediction of LLNM, showing good discrimination and calibration in independent validation cohorts (46–48). In this study, model validation demonstrated good discrimination ability. This model demonstrates high predictive accuracy, but the PPV is relatively low, which may be attributable to the low prevalence of the disease in the dataset. This finding suggests that for patients identified as positive by the model, increased vigilance is warranted, and confirmation should only be made after thorough diagnostic evaluation. The calibration analysis indicated that the predicted probabilities closely matched the observed probabilities, thus validating the model’s reliability. Through DCA, the clinical utility of the model was quantified, using this model could bring net benefits to patients. This predictive model is primarily intended for postoperative risk stratification. For low-risk patients, the frequency of imaging follow-up may be reduced, whereas high-risk patients should receive more intensive follow-up strategies to remain vigilant for possible occult metastasis.

This study still had some limitations and shortcomings. Firstly, as it was a retrospective study, it could not completely eliminate potential selection biases, which limited the in-depth exploration of the interrelationships among risk factors. Secondly, although the sample size was relatively large, this study came from a single center and lacked multicenter validation, which might limit the external general applicability of the research results and failed to fully reflect the actual situations of different populations and regions. Multicenter large-scale prospective studies may be able to further clarify the interactions among the complex factors influencing LLNM.

## 5 Conclusion

This study confirmed that post-mETE was an independent risk factor for LLNM, while ant-mETE had little impact on LLNM. Compared to middle region tumors and lower pole tumors, upper pole tumors were more likely to develop LLNM. The nomogram model developed in this study could effectively assess the risk of LLNM in PTC patient. For high-risk patients with high nomogram scores, clinicians may consider adopting stricter follow-up management or adjuvant radioactive iodine therapy.

## Data Availability

The studies involving humans were approved by Institutional Review Board of Qilu Hospital. The studies were conducted in accordance with the local legislation and institutional requirements. The participants provided their written informed consent to participate in this study.
