# Supplementary material for: Posterior minimal extrathyroidal extension as an independent risk factor for lateral lymph node metastasis in papillary thyroid carcinoma: a retrospective study based on a nomogram prediction model
Source: Front Endocrinol (Lausanne). 2026 Jun 17;17:1853174. doi: 10.3389/fendo.2026.1853174 (PMC13318688; doi:10.3389/fendo.2026.1853174)
Supplement: Supplementary file 1 [file Table1.docx]

Supplementary Material

# Supplementary Tables

**Supplementary Table 1.** Univariate analysis of risk factors for LLNM after 1:1 propensity score matching based on CLNM.

|  |  | **Lateral Lymph Node Metastasis** | | | |  |
| --- | --- | --- | --- | --- | --- | --- |
| **Characteristics** | **Total**  **(n=600)** | **Yes**  **(n=300)** | | **No**  **(n=300)** | | ***P* value** |
| **Age** |  |  |  |  |  | 0.656 |
| <55 | 504 | 250 (83.33%) | | 254 (84.67%) | |  |
| ≥55 | 96 | 50 (16.67%) | | 56 (15.33%) | |  |
| **Sex** |  |  |  |  |  | 0.531 |
| Female | 423 | 208 (69.33%) | | 215 (71.67%) | |  |
| Male | 177 | 92 (30.67%) | | 85 (28.33%) | |  |
| **BMI** |  |  |  |  |  | 0.806 |
| <25 | 327 | 165 (55.00%) | | 162 (54.00%) | |  |
| ≥25 | 273 | 135 (45.00%) | | 138 (46.00%) | |  |
| **Maximum Tumor Size** |  |  |  |  |  | 0.869 |
| ≤1cm | 346 | 172 (57.33%) | | 174 (58.00%) | |  |
| >1cm | 254 | 128 (42.67%) | | 126 (42.00%) | |  |
| **Bilateral** |  |  |  |  |  | 0.791 |
| Yes | 185 | 94 (31.33%) | | 91 (30.33%) | |  |
| No | 415 | 206 (68.67%) | | 209 (69.67%) | |  |
| **Multifocality** |  |  |  |  |  | 0.595 |
| Yes | 184 | 95 (31.67%) | | 89 (29.67%) | |  |
| No | 416 | 205 (68.33%) | | 211 (70.33%) | |  |
| **HT** |  |  |  |  |  | 0.125 |
| Yes | 173 | 78 (26.00%) | | 95 (31.67%) | |  |
| No | 427 | 222 (74.00%) | | 205 (68.33%) | |  |
| **NG** |  |  |  |  |  | 0.682 |
| Yes | 271 | 138 (46.00%) | | 133 (44.33%) | |  |
| No | 329 | 162 (54.00%) | | 167 (55.67%) | |  |
| **Number of CLNM** |  |  | |  | | <0.001 |
| 0 | 173 | 47 (15.67%) | | 126 (42.00%) | |  |
| 1 | 121 | 53 (17.67%) | | 68 (22.67%) | |  |
| ≥2 | 306 | 200 (66.66%) | | 106 (35.33%) | |  |
| **Tumor Location** |  |  |  |  |  | 0.948 |
| Middle | 222 | 111 (37.00%) | | 111 (37.00%) | |  |
| Upper | 253 | 125 (41.67%) | | 128 (42.67%) | |  |
| Lower | 125 | 64 (21.33%) | | 61 (20.33%) | |  |
| **mETE** |  |  |  |  |  | 0.898 |
| Without | 116 | 60 (20.00%) | | 56 (18.67%) | |  |
| Anterior | 206 | 101 (33.67%) | | 105 (35.00%) | |  |
| Posterior | 278 | 139 (46.33%) | | 139 (46.33%) | |  |
| **BRAF V600E Mutation** |  |  | |  | | 0.223 |
| Positive | 404 | 209 (69.67%) | | 195 (65%) | |  |
| Negative | 196 | 91 (30.33%) | | 105 (35) | |  |

PTC, papillary thyroid carcinoma; LLNM, lateral lymph node metastasis; BMI, body mass index; HT hashimoto's thyroiditis; NG, nodular goiter; CLNM, central lymph node metastasis; mETE, minimal extrathyroidal extension

**Supplementary Table 2.** Univariate analysis of risk factors for LLNM after 1:1 propensity score matching based on tumor location.

|  |  | **Lateral Lymph Node Metastasis** | | | |  |
| --- | --- | --- | --- | --- | --- | --- |
| **Characteristics** | **Total**  **(n=582)** | **Yes**  **(n=291)** | | **No**  **(n=291)** | | ***P* value** |
| **Age** |  |  |  |  |  | 0.640 |
| <55 | 496 | 246 (84.54%) | | 250 (85.91%) | |  |
| ≥55 | 86 | 45 (15.46%) | | 41 (14.09%) | |  |
| **Sex** |  |  |  |  |  | 0.649 |
| Female | 411 | 208 (71.48%) | | 203 (69.76%) | |  |
| Male | 171 | 83 (28.52%) | | 88 (30.24%) | |  |
| **BMI** |  |  |  |  |  | 0.357 |
| <25 | 333 | 161 (55.33%) | | 172 (59.11%) | |  |
| ≥25 | 249 | 130 (44.67%) | | 119 (40.89%) | |  |
| **Maximum Tumor Size** |  |  |  |  |  | 0.271 |
| ≤1cm | 349 | 168 (57.73%) | | 181 (62.20%) | |  |
| >1cm | 233 | 123 (42.27%) | | 110 (37.80%) | |  |
| **Bilateral** |  |  |  |  |  | 0.323 |
| Yes | 179 | 84 (28.87%) | | 95 (32.65%) | |  |
| No | 403 | 207 (71.13%) | | 196 (67.35%) | |  |
| **Multifocality** |  |  |  |  |  | 0.165 |
| Yes | 161 | 88 (30.24%) | | 73 (25.09%) | |  |
| No | 421 | 203 (69.76%) | | 218 (74.91%) | |  |
| **Hashimoto’s Thyroiditis** |  |  |  |  |  | 0.561 |
| Yes | 140 | 73 (25.09%) | | 67 (23.02%) | |  |
| No | 442 | 218 (74.91%) | | 224 (76.98%) | |  |
| **Nodular Goiter** |  |  |  |  |  | 0.678 |
| Yes | 277 | 141 (48.45%) | | 136 (46.74%) | |  |
| No | 305 | 150 (51.55%) | | 155 (53.26%) | |  |
| **Number of CLNM** |  |  | |  | | 0.797 |
| 0 | 118 | 61 (20.96%) | | 57 (19.59%) | |  |
| 1 | 124 | 64 (21.99%) | | 60 (20.62%) | |  |
| ≥2 | 340 | 166 (57.05%) | | 174 (59.79%) | |  |
| **Tumor Location** |  |  |  |  |  | <0.001 |
| Middle | 213 | 101 (34.71%) | | 112 (38.48%) | |  |
| Upper | 203 | 141 (48.45%) | | 62 (21.31%) | |  |
| Lower | 166 | 49 (16.84%) | | 117 (40.21%) | |  |
| **Minimal Extrathyroidal Extension** |  |  |  |  |  | 0.190 |
| Without | 111 | 60 (20.62%) | | 51 (17.53%) | |  |
| Anterior | 184 | 82 (28.18%) | | 102 (35.05%) | |  |
| Posterior | 287 | 149 (51.20%) | | 138 (47.42%) | |  |
| **BRAF V600E Mutation** |  |  | |  | | 0.470 |
| Positive | 406 | 207 (71.13%) | | 199 (68.38%) | |  |
| Negative | 176 | 84 (28.27%) | | 92 (31.62%) | |  |

PTC, papillary thyroid carcinoma; LLNM, lateral lymph node metastasis; BMI, body mass index; HT hashimoto's thyroiditis; NG, nodular goiter; CLNM, central lymph node metastasis; mETE, minimal extrathyroidal extension

**Supplementary Table 3.** Univariate analysis of risk factors for LLNM after 1:1 propensity score matching based on mETE.

|  |  | **Lateral Lymph Node Metastasis** | | | |  |
| --- | --- | --- | --- | --- | --- | --- |
| **Characteristics** | **Total**  **(n=554)** | **Yes**  **(n=277)** | | **No**  **(n=277)** | | ***P* value** |
| **Age** |  |  |  |  |  | 0.648 |
| <55 | 462 | 229 (82.67%) | | 233 (84.12%) | |  |
| ≥55 | 92 | 48 (17.33%) | | 44 (15.88%) | |  |
| **Sex** |  |  |  |  |  | 0.266 |
| Female | 388 | 200 (72.20%) | | 188 (67.87%) | |  |
| Male | 166 | 77 (27.80%) | | 89 (32.13%) | |  |
| **BMI** |  |  |  |  |  | 0.496 |
| <25 | 292 | 150 (54.15%) | | 142 (51.26%) | |  |
| ≥25 | 262 | 127 (45.85%) | | 135 (48.74%) | |  |
| **Maximum Tumor Size** |  |  |  |  |  | 0.668 |
| ≤1cm | 313 | 154 (55.60%) | | 159 (57.40%) | |  |
| >1cm | 241 | 123 (44.40%) | | 118 (42.60%) | |  |
| **Bilateral** |  |  |  |  |  | 0.780 |
| Yes | 165 | 84 (30.32%) | | 81 (29.24%) | |  |
| No | 389 | 193 (69.68%) | | 196 (70.76%) | |  |
| **Multifocality** |  |  |  |  |  | 0.520 |
| Yes | 171 | 89 (32.13%) | | 82 (29.60%) | |  |
| No | 383 | 188 (67.87%) | | 195 (70.40%) | |  |
| **Hashimoto’s Thyroiditis** |  |  |  |  |  | 0.281 |
| Yes | 139 | 75 (27.08%) | | 64 (23.10%) | |  |
| No | 415 | 202 (72.92%) | | 213 (76.90%) | |  |
| **Nodular Goiter** |  |  |  |  |  | 0.671 |
| Yes | 265 | 135 (48.74%) | | 130 (46.93%) | |  |
| No | 289 | 142 (51.26%) | | 147 (53.07%) | |  |
| **Number of CLNM** |  |  | |  | | 0.426 |
| 0 | 115 | 61 (22.02%) | | 54 (19.49%) | |  |
| 1 | 120 | 54 (19.49%) | | 66 (23.83%) | |  |
| ≥2 | 319 | 162 (58.49%) | | 157 (56.68%) | |  |
| **Tumor Location** |  |  |  |  |  | 0.885 |
| Middle | 203 | 102 (36.82%) | | 101 (36.46%) | |  |
| Upper | 229 | 112 (40.43%) | | 117 (42.24%) | |  |
| Lower | 122 | 63 (22.75%) | | 59 (21.30%) | |  |
| **Minimal Extrathyroidal Extension** |  |  |  |  |  | 0.007 |
| Without | 118 | 51 (18.41%) | | 67 (24.19%) | |  |
| Anterior | 181 | 80 (28.88%) | | 101 (36.46%) | |  |
| Posterior | 255 | 146 (52.71%) | | 109 (39.35%) | |  |
| **BRAF V600E Mutation** |  |  | |  | | 0.283 |
| Positive | 364 | 188 (67.87%) | | 176 (63.54%) | |  |
| Negative | 190 | 89 (32.13%) | | 101 (36.46%) | |  |

PTC, papillary thyroid carcinoma; LLNM, lateral lymph node metastasis; BMI, body mass index; HT hashimoto's thyroiditis; NG, nodular goiter; CLNM, central lymph node metastasis; mETE, minimal extrathyroidal extension
